# Evaluating indices of traditional ecological knowledge: a methodological contribution

**DOI:** 10.1186/1746-4269-2-21

**Published:** 2006-04-25

**Authors:** Victoria Reyes-García, Vincent Vadez, Susan Tanner, Thomas McDade, Tomás Huanca, William R Leonard

**Affiliations:** 1 ICREA and ICTA, Universitat Autònoma de Barcelona, 08193 Bellaterra, Barcelona, Spain and Sustainable International Development Program, Brandeis University, Waltham, MA 02454, USA; 2 Sustainable International Development Program, Heller School for Social Policy and Management, Brandeis University, Waltham, MA 02454, USA; 3 Department of Anthropology, Northwestern University, Evanston, IL 60208, USA

## Abstract

**Background:**

New quantitative methods to collect and analyze data have produced novel findings in ethnobiology. A common application of quantitative methods in ethnobiology is to assess the traditional ecological knowledge of individuals. Few studies have addressed reliability of indices of traditional ecological knowledge constructed with different quantitative methods.

**Methods:**

We assessed the associations among eight indices of traditional ecological knowledge from data collected from 650 native Amazonians. We computed Spearman correlations, Chronbach's alpha, and principal components factor analysis for the eight indices.

**Results:**

We found that indices derived from different raw data were weakly correlated (rho<0.5), whereas indices derived from the same raw data were highly correlated (rho>0.5; p < 0.001). We also found a relatively high internal consistency across data from the eight indices (Chronbach's alpha = 0.78). Last, results from a principal components factor analysis of the eight indices suggest that the eight indices were positively related, although the association was low when considering only the first factor.

**Conclusion:**

A possible explanation for the relatively low correlation between indices derived from different raw data, but relatively high internal consistency of the eight indices is that the methods capture different aspects of an individual's traditional ecological knowledge. To develop a reliable measure of traditional ecological knowledge, researchers should collect raw data using a variety of methods and then generate an aggregated measure that contains data from the various components of traditional ecological knowledge. Failure to do this will hinder cross-cultural comparisons.

## Background

Methodological contributions are essential in any branch of science and many researchers have shown concern with a perceived lack of methodological advances in contemporary ethnobiology [1]. Ethnobiology has often been criticized for focussing on list making and lacking methodological rigor. Before the mid-1950s, research in ethnobiology was primarily descriptive, but by the mid 1980s, researchers had already incorporated a variety of quantitative methods of data collection and data analysis. At present, quantitative methods in ethnobiology -shared with other biological, social, and linguistic sciences- are proliferating [2,3].

Although the use of quantitative methods is becoming common in ethnobiology, we still lack studies assessing the reliability of data collected with different methods. For example, researchers have applied quantitative methods to assess the traditional ecological knowledge of individuals and groups, but the methods used to collect and to transform the data vary across studies. Thus, to collect raw data on the traditional ecological knowledge of individuals, researchers have used open-ended interviews [4], structured questionnaires [5], specimen identification [6], and direct observations of participant's behavior [7]. Once researchers collect the raw information, they also use different methods to construct indices or summary measures of traditional ecological knowledge. Common methods to construct such indices include cultural consensus [8], matching of responses with ecological data [9], and diversity indices [10]. What we lack now are studies that assess the reliability of the different indices that presumably proxy for the same phenomena or the traditional ecological knowledge of a person.

Assessing the correspondence of data collected and transformed with different methods should contribute to ethnobiological studies, and particularly to studies of traditional ecological knowledge, in two ways. First, assessing the correspondence of data collected and transformed with different methods will enhance cross-cultural comparisons. For example, quantitative studies about the determinants of the loss of traditional ecological knowledge show conflicting results across sites. Some studies suggest that socio-economic changes do not decrease traditional ecological knowledge [6], others suggest that only certain socioeconomic changes decrease traditional ecological knowledge [11,12], and still others suggest that integration into the market economy through activities based on the natural environment could accelerate the acquisition of ecological knowledge [13]. Divergent conclusions might reflect the use of different methods because different methods might have captured dimensions of traditional ecological knowledge that do not necessarily overlap [14]. Cross-cultural comparisons about the acquisition, transmission, and loss of traditional ecological knowledge will be enhanced when researchers use methods with acceptable reliability.

The second reason why assessing the correspondence of data collected and transformed with different methods might enhance ethnobiological research is that such studies might help identify methods apt to measure various aspects of traditional ecological knowledge. Researchers have presented many definitions of traditional ecological knowledge. A universal, measurable definition is not likely because traditional ecological knowledge comprises many fields (i.e., plants, soils) and many dimensions (i.e., theory, practice, beliefs). Specific methods appropriate to capture information in one field or dimension might not be appropriate for another field or dimension. For example, observing people's use of plants might shows us their ability to apply traditional knowledge, but it says little about the individual's theoretical knowledge of the same plants. By studying the correspondence of data collected and analyzed with different methods, we might be able to identify the kind of knowledge captured by each method.

The goal of this paper is to assess the correspondence between eight indices of traditional ecological knowledge. We used four different methods to collect raw data from 650 Tsimane' Amazonians in Bolivia, and from that data we constructed eight indices. The study forms part of a long-term research to measure the effect of markets on the quality of life of indigenous peoples [15].

The information used in this paper is unique in at least three ways. First, the data were collected by a multidisciplinary team of anthropologists, biologists, and agronomists who lived in the study area during 18 consecutive months (May 2002-November 2003). This contrasts with studies of traditional ecological knowledge that have been done by authors from a single discipline or over short periods of time. Long periods of research and multidisciplinary teams, should enhance the quality of the data collected. Second, the sample size (n = 650) was more than five times larger than the average sample size of the typical study measuring individual traditional ecological knowledge [16]. A larger sample size, though only from one culture, should enhance the confidence we can attach to the results of the comparison of methods.

Third, we distinguished between the theoretical and the practical dimensions of traditional ecological knowledge and collected information on both dimensions. Knowledge refers to the theoretical dimension, or intellectual ability, such as the ability to name plants. Skill refers to the ability to put knowledge into practice. For example, some people may know the potential uses of a plant, but they may not know how to use the plant. By including more than one dimension of traditional ecological knowledge, we can test whether the two dimensions reflect an underlying construct. This contrasts with other studies that focus on either theoretical knowledge or practical skills.

## Methods

### The people

We conducted research among the Tsimane', a native Amazonian population of about 8,000 people living in about 100 villages in the department of Beni, Bolivia. Recent publications [3,17-19] provide ethnographic information on the Tsimane', including descriptions of their traditional ecological knowledge [20-23]. The Tsimane' provide an apt case to study the measure of traditional ecological knowledge of plants because they display a level of ethnobotanical knowledge comparable to other groups in the region [24,25], and because they share the knowledge [26].

### Sample size

We collected information from all the adults (>16 years of age) from 13 villages along the Maniqui River. To select the 13 villages for the study, we used village distance from the town of San Borja, the regional commercial center (population ~19,000). We interviewed all people over the age of 16 because, at this age Tsimane' adolescents start forming their own households and enter adulthood. A total of 650 adults from 13 Tsimane' villages participated in the study, but only 375 (or 48% of the 650) provided information for all the methods of data collection. Although we obtained complete data only for about half of the sample, the results are representative of the villages surveyed. We refer to the sample with raw data for all the methods as "permanent sample", to differentiate it form the "non-permanent sample", or people who did not provided raw information for one or more methods. Because the goal of the article is to compare results across methods, we mostly use data from the permanent sample for the analysis. We use the additional information provided by the non-permanent sample to test whether the results hold across samples. The permanent sample was almost evenly split between women (n = 201, or 53.6%) and men (n = 174). The average age of the person in the permanent sample was 35.4 years of age (sd = 15.1).

### Methods to collect raw data

For each participant, we collected four sets of raw data. For three of the four sets of raw data, we collected information over three months, and for the last method we conducted weekly interviews on the uses of plants over a year (October 2002-October 2003). We have given detailed explanations of most of the methods presented here in previous publications [8,23,26,27], so here we summarize those methods. Table 1 contains a summary of the construction of the eight indices, indicating the method used to collect the raw data and the method used to transform the raw data.

**Table 1:** Definition of methods to collect raw data and construct indices

	**Method to collect raw data**	**Method to construct index**	**Index**	**Definition**
THEORETICAL DIMENSION	(1) Multiple-choice task on uses of wild plants	Cultural consensus	*Cultural knowledge of uses*	% of individual questions coinciding with the most frequent response in the group
		Matching with experts	*Agreement with experts*	% of individual questions matching with the answers from elders in the group
	(2) Multiple-choice task on ecology of wild plants	Cultural consensus	*Ecological cultural knowledge*	% of individual questions coinciding with the most frequent response in the group
		Matching ecological data	*Ecological knowledge*	Number of responses on plant ecology matching textbook information.

PRACTICAL DIMENSION	(3) Interview of reported use of plants	Data aggregation	*Average plants used*	Average number of plants brought to the household per day
		Data aggregation	*Total plants used*	Number of plants brought to the household/entire search period
		Richness index	*Total species used*	Total number of different species brought to the household/entire research period
	(4) Questionnaire on skills	Data aggregation	*Skills using plants*	Self-reported number of plant-made items that the participant reported knowing how to make

Multiple-choice. We constructed two different multiple-choice tasks to measure knowledge of local plants. We selected multiple-choice tasks to measure theoretical knowledge because multiple-choice tasks are the most common method used to measure traditional ecological knowledge [16]. Additionally, when working with indigenous populations, multiple-choice tasks are more reliable than other types of structured questionnaires, such as paired comparisons and triads [27].

To construct the first task, we randomly selected 21 plants from a list of Tsimane' useful plants [8]. The task consisted of asking participants whether the 21 plants selected could be used for construction, firewood, food, medicine, or other ends. For each plant, participants could choose none, one, or more potential uses. We collected the information in the form of a matrix with the names of the plants on the X-axis, and the possible uses on the Y-axis. We coded affirmative answers as one and negative answers as zero.

The second multiple-choice task had to do with the ecology of plants. We asked about biological characteristics of ten plants randomly chosen from the list of the wild plants mentioned before, and presented the participants with three possible answers from which they could chose only one. For example, we asked: "Which is the color of the mahogany flower? a) red, b) green, or c) white". If participants were not sure about the answer we asked them to provide their best guess. Questions related to the habitat where the plant is found, phenotypic traits (e.g., color of the flower), and the ecology of the plants (i.e., flowering and ripping times).

Interviews about the uses of plants. To capture the practical dimension of traditional ecological knowledge, we used interviews to measure daily uses of plants. Every week, on a day chosen at random, we visited all households during a three-hour block falling from 7am until 7pm; we also chose the blocks of time at random. During those visits, we asked each adult present in the household to name all of the wild plants the person had brought to the household during the previous 24 hours. We only included adults present in the household at the time of the interview. Absent adults were coded as missing.

Questionnaire on skills. To collect information on participant's practical abilities we used a questionnaire on self-reported skills about crafting objects from wild plants. We asked participants whether they had ever made on their own 18 objects from a list of 15 different plants [12]. Three key informants helped to create the list of objects. The list included nine objects that are more commonly made by men and nine that are more commonly made by women. Each list also included six items that key informants considered easy to make, six items they considered of medium difficulty, and six items they considered difficult to make.

### Indices of traditional ecological knowledge

We transformed data collected with the above methods to obtain eight indices of individual traditional ecological knowledge.

#### Cultural knowledge of uses

We analyzed data from the multiple-choice task on uses of plants with the cultural consensus method [28]. To calculate the index of *cultural knowledge of plant uses*, for each individual we computed the proportion of individual questions coinciding with the most frequent response in the group.

#### Agreement with experts

With data from the multiple-choice task on uses of plants we also generated an index measuring the degree of individual agreement with the experts of the group. To do so, we first generated an "answer key" to the multiple-choice task using answers from individuals over 55 years of age. We considered elders as experts because studies suggest that traditional ecological knowledge bears a positive association with age [4,29,30]. We then compared the participants' responses to the experts' answers.

#### Ecological cultural knowledge

We also used the cultural consensus method to analyze data on plant ecology from the multiple-choice task. We called the proportion of individual questions coinciding with the most frequent response of the group *ecological cultural knowledge*.

#### Ecological knowledge

We used data from the multiple-choice task on plant ecology to generate an index of *ecological knowledge*. For each participant, we added the number of times a response matched ecological data from the area. For example, Killeen [31] reports that the color of the mahogany flower is white, so we consider white the correct response to the question "What is the color of the mahogany flower?" Ecological information from the area was obtained from Hinojosa [32,33] and Killeen and colleagues [31].

#### Average plants used

We used data from repeated interviews on uses of plants to generate an index measuring the average number of wild plants used by a person on a given day. We generated the index by adding the number of different plants each person brought during the entire period of research and dividing it by the number of observations for each person [34].

#### Total plants used

We also used data from interviews on uses of plants to generate an index measuring the total number of plants used by a person over the research period [10]. To construct such an index, we added all the plants the person reported bringing into the household during the research period, independent of the number of interviews done to the person.

#### Total species used

This index captures the richness or diversity of species used by a person over the research period [14]. To construct the index, we counted the total number of different species brought home by a participant during the duration of the study, independent of the number of interviews in which the person participated.

#### Skills using plants

We used responses to the questionnaire on skills to construct another index of the practical dimension of traditional ecological knowledge. To calculate the index, we summed the number of goods each person reported knowing how to make from the skill questionnaire.

### Data analysis

We test for the normality of the sample using the skewness and kurtosis tests of normality. Since our data were not normally distributed, we used non-parametric statistics. We first compare results across the permanent and non-permanent samples using Mann-Whitney two-sample tests. We then computed the Spearman rank correlation coefficients (rho) between the eight different indices. To test the internal consistency of the eight indices, we computed the Chronbach's alpha and the principal components factors for the eight indices. The Chronbach's alpha computes the average inter-correlation between all the items in a scale. Since our indices had different units, before computing the Chronbach's alpha we standardized all the indices (mean = 0 and standard deviation = 1).

## Results

Table 2 shows the descriptive statistics for the eight indices. Results from a Mann-Whitney test show that there was no statistically significant difference between three out of the four indices of theoretical traditional ecological knowledge between participants who provided answers for all the methods and participants who did not provided answers for one or more methods. We also found that the indices of practical traditional ecological knowledge and the index that measures agreement with experts were significantly higher for the permanent sample than for the non-permanent sample.

**Table 2:** Comparison of indices of traditional ecological knowledge between two samples (permanent and non-permanent).

	**Permanent Sample (n = 375)**				**Non-permanent Sample**					**Mann-Whitney two-sample**
**Index**	**Mean**	**Std**	**Median**	**Variance**	**Obs**	**Mean**	**Std**	**Median**	**Variance**	**Prob>| z| **

(1) *Cultural knowledge of uses*	0.58	0.20	0.56	0.04	155	0.55	0.19	0.57	0.03	0.23
(1) *Agreement with experts*	13.61	3.08	14	9.47	155	12.66	3.37	13.2	11.3	0.005
(2) *Ecological cultural knowledge*	0.55	0.18	0.57	0.03	110	0.55	0.18	0.56	0.03	0.96
(2) *Ecological knowledge*	5.48	1.49	6	2.21	110	5.53	1.64	5	2.69	0.95
(3) *Average plants used*	0.14	0.15	0.09	0.02	275	0.08	0.13	0	0.01	<0.0001
(3) *Total plants used*	2.58	3.66	1	13.4	275	0.90	1.75	0	3.05	<0.0001
(3) *Total species used*	0.87	1.06	1	1.13	275	0.49	0.81	0	0.65	<0.0001
(4) *Skills using plants*	8.11	3.02	8	9.1	160	7.47	3.47	8	12.06	0.03

(1) Data collected with multiple-choice task on uses of wild plants; (2) Data collected with multiple-choice task on ecology of wild plants; (3) Data collected with interviews of reported use of plants; (4) Data collected with questionnaire on skills

Table 3 shows the results of Spearman correlations among the eight indices. We found high and positive correlation coefficients between variables derived from the same raw data. For example, the correlation coefficient between the indices of *cultural knowledge of uses *and *agreement with experts *was 0.85 (p < 0.001) and the correlation coefficient between *ecological cultural knowledge *and *ecological knowledge *was 0.55 (p < 0.001).

**Table 3:** Spearman correlations among eight indices of traditional ecological knowledge (n = 375).

		THEORETICAL DIMENSION			PRACTICAL DIMENSION			
		*(1)*	*(2)*	*(2)*	*(3)*	*(3)*	*(3)*	*(4)*

	**Index**	*Agreement with experts*	*Ecological cultural knowledge*	*Ecological knowledge*	*Average plants used*	*Total plants used*	*Total species used*	*Skills using plants*

**THEORETICAL**	*(1)Cultural knowledge of uses*	0.855***	0.050	0.179***	0.385***	0.404***	0.281***	0.174***
	*(1)Agreement with experts*		0.136***	0.319***	0.412***	0.483***	0.393***	0.217***
	*(2)Ecological cultural knowledge*			0.556***	0.102**	0.139***	0.097*	0.148***
	*(2)Ecological knowledge*				0.146***	0.212***	0.241***	0.140***

**PRACTICAL**	*(3)Average plants used*					0.952***	0.557***	0.243***
	*(3) Total plants used*						0.659***	0.221***
	*(3) Total species used*							0.104**

Notes: *, **, and *** significant at the ≤ 10%, ≤ 5% or ≤ 1% level. (1) Data collected with multiple-choice task on uses of wild plants; (2) Data collected with multiple-choice task on ecology of wild plants; (3) Data collected with interviews of reported use of plants; (4) Data collected with questionnaire on skills

We found low correlation coefficients between indices derived from raw data collected with different methods. For example, the index of *ecological knowledge *correlated in a significant and positive way with the indices of *cultural knowledge of uses *and *agreement with experts*, but the correlation coefficients were low (rho<0.35). The correlation of *ecological cultural knowledge *with the indices from raw data from the first multiple-choice task was even lower (rho<0.15 for *agreement with expert*) or non existent (with *cultural knowledge of uses*). We found similar results when comparing measures of practical skill. The three indices derived from interviews on daily uses of plants correlated in a statistically significant and positive way with one another (rho>0.55; p < 0.001). The correlation coefficients of those indices with the index of *skills using plants *were below 0.25 (p < 0.001).

Last, we found that the indices of theoretical knowledge also correlated with the indices of practical skills, but all the correlation coefficients were low (rho<0.50). The index of theoretical knowledge that showed the lowest correlation coefficient with the indices of practical skills was *ecological cultural knowledge *(rho<0.15). The index of practical skills that showed the lowest correlation with the indices of theoretical knowledge was *skills using plants *(rho<0.22).

To test the robustness of the associations, we ran the same correlations but using the total sample, i.e. including the permanent and the non-permanent samples (not shown). We found similar coefficients and levels of statistical significance for all the indices.

To test the internal consistency of the eight indices, we calculated the Chronbach's alpha of the different indices. We found that the Chronbach's alpha for the four indices of theoretical knowledge and practical skills were 0.69 and 0.72. We then computed the Chronback's alpha for the eight indices together and found that the eight items were positively related. The Chronbach's alpha of the eight indices was 0.78, higher than the Chronbach's alpha coefficients of indices of theoretical knowledge and indices of practical skills.

As a last test of reliability, we ran a principal components factor analysis of the eight indices (Table 4). As in previous tests, we found that the eight indices were positively related, although the association was low when considering only the first factor. The first factor of the principal component analysis had an eigenvalue of 3.38, and explained about 42% of the variation in our data. The first three factors explained 75% of variation in data.

**Table 4:** Principal component factor analysis of eight indices of traditional ecological knowledge (n = 375)

Variable	Factor loadings			Uniqueness
	1	2	3	

*Cultural knowledge of uses*	0.73723	-0.18771	0.556	0.11
*Agreement with experts*	0.79549	-0.04522	0.50679	0.11
*Ecological cultural knowledge*	0.3361	0.82299	-0.11305	0.20
*Ecological knowledge*	0.45919	0.75426	-0.02407	0.22
*Average plants used*	0.77752	-0.2847	-0.33478	0.20
*Total plants used*	0.85005	-0.20598	-0.34651	0.11
*Total species used*	0.69175	-0.1106	-0.41778	0.33
*Skills using plants*	0.2757	0.08834	0.28966	0.83

We found that the two indices derived from the multiple-choice task in uses of plants and the three indices derived from interviews about the daily use of plants loaded strongly and positively on the first factor and negatively on the second factor. We also found that the two indices derived from the multiple-choice questions about ecology loaded weakly on the first factor, but positively and strongly on the second factor. Last, the index of *skills using plants *loaded weakly on three factors. In fact, the index of *skills using plants *had the lowest factor loadings (0.27), meaning that 83% of the variance of the index remained unexplained by the first three components.

## Discussion and conclusions

In this article we have tested the degree of correspondence among eight indices of traditional ecological knowledge from different methods. We found that indices derived from different raw data were weakly correlated, but we also found that all the indices reflected a single underlying construct, as shown by the results of Chronbach's alpha and a principal components factor analyses. Why did the indices show low partial correlation coefficients but a high degree of overall agreement?

We can think of two possible explanations for the low correlations found when comparing raw data collected with different methods. First, it is possible that some methods are more prone to bias and random measurement error than others. For example, some of the tasks may have been harder to understand than other tasks. Data collected with tasks harder to understand may contain more random measurement error than data collected with tasks that are easier to understand. Furthermore, each task might be subject to its own type of random measurement error. For example, data collected through weekly interviews may be subject to informant's recall error; participants might neglect to mention less significant plants brought to the household, thereby underestimating the number of plants used.

A second possible explanation for the low correlations found when comparing raw data collected with different methods is that the methods measured different aspects of traditional ecological knowledge. For example, previous research comparing theoretical knowledge and practical uses of plants suggests that the two types of knowledge do not correlate well [4,14,22,35]. This is a logical explanation for the generally low correlation coefficients that we found between indices that proxy for the theoretical and practical dimensions of traditional ecological knowledge. A similar explanation applies for the lack of correlation between the variables *cultural knowledge of uses *and *ecological cultural knowledge*. The low correlation between both variables cannot be attributed to random measurement error because data were collected and transformed using the same methods. However, the lack of correlation between the two variables could reflect differences in the domain of traditional ecological knowledge that they capture. The first variable, *cultural knowledge of uses*, measures knowledge that is culturally specific, whereas the second variable, *ecological cultural knowledge*, measures the correspondence of local knowledge with scientifically validated ecological knowledge.

If different methods measure different aspects of traditional ecological knowledge, why then do results of Chronbach's alpha suggest the existence of one underlying construct? Recall that when we computed Chronbach's alpha for the eight indices we found that it was higher than the Chronbach's alpha for the indices of theoretical knowledge and of practical skills separately. Because we found a relatively high association between the eight indices, we can assume that they reflect the same underlying phenomenon (i.e., traditional ecological knowledge), but because we are trying to measure a complex phenomenon, composed of many dimensions and domains, it is reasonable to think that comparisons of the different components do not show high correlations.

While a high value for Cronbach's alpha indicates good internal consistency of the indices used, it does not mean that the construct analyzed is uni-dimensional. Results from the principal component factor analysis help us to identify some of the different components or dimensions of traditional ecological knowledge. The principal component factor analysis suggests the presence of at least three components of traditional ecological knowledge. The first component refers to knowledge that is culturally constructed. This component was captured by the five indices measuring uses of plants, or the indices that loaded strongly and positively on the first factor and loaded negatively on the second factor. The second component referred to ecological knowledge that could have been scientifically validated. This component was captured by the two indices measuring ecological knowledge, which loaded weakly on the first factor, and positively and strongly on the second factor. The third component reflected the participant's practical skills to use plants. The third component was not well explained by our data, possibly because we only constructed one index for practical skills.

As noted in the introduction, researchers have suggested that traditional ecological knowledge comprises many fields and many dimensions of knowledge. The results of our empirical work confirm the idea that specific methods appropriate to capture information in one field or dimension of traditional ecological knowledge might not be appropriate to capture information in another field or dimension. For example, none of the indices of theoretical traditional ecological knowledge is highly associated with any of the indices of practical skills. Therefore, methods designed to capture theoretical knowledge will not capture accurately the ability to use that knowledge.

A last point requires discussion. When comparing the permanent and non-permanent samples, we found that all the indices of practical knowledge from the permanent sample were significantly higher than the indices of practical knowledge from the non-permanent sample, but that only one of the indices of theoretical knowledge, *agreement with experts*, was higher than the index from the non-permanent sample. The finding suggests that living outside a community does not affect the individual theoretical knowledge, but it does affect the individual's practical abilities to use the knowledge. The finding has implications for sample selection in future studies attempting to measure individual practical knowledge. The selection of a sample that lives permanently in the village might raise the estimates of individual's practical knowledge.

We conclude with two suggestions to improve future research. First, we need further studies assessing the reliability of different methods. Researchers have used many quantitative methods to collect data and construct indices of traditional ecological knowledge, but they have not paid enough attention to the reliability of the various methods used. To develop a metric of individual traditional ecological knowledge that can be used in cross-cultural research, we need to assess the reliability of methods of data collection. Second, we recommend the development of a comprehensive measure of traditional ecological knowledge. Traditional ecological knowledge is a complex construct, so developing a comprehensive measure of traditional ecological knowledge will require the use of a variety of methods to collect data on knowledge, skills, and beliefs of different fields of ecological knowledge.

## Competing interests

The author(s) declare that they have no competing interests.
